# A Holistic Nursing Surveillance Decision Support System for Postoperative Pulmonary Complications After Abdominal Surgery: A Retrospective Cohort Study

**DOI:** 10.3390/healthcare14081083

**Published:** 2026-04-18

**Authors:** Se Young Kim, Dong Hyun Lim, Dae Ho Kim, Ok Ran Jeong

**Affiliations:** 1 Department of Nursing, Changwon National University, Changwon-si 51140, Republic of Korea; sarakimk@changwon.ac.kr; 2 School of Computing, Gachon University, Seongnam-si 13120, Republic of Korea; ansxorwlstnr@gachon.ac.kr (D.H.L.); ikimdh91@gachon.ac.kr (D.H.K.)

**Keywords:** postoperative pulmonary complications (PPCs), decision support systems, clinical, nursing care, electronic medical records (EMRs), deep learning

## Abstract

**Background/Objectives**: Postoperative pulmonary complications (PPCs) following abdominal surgery are associated with prolonged hospitalization, delayed recovery, and increased mortality. Because nursing surveillance is essential for early detection and timely intervention, this study aimed to develop a holistic nursing surveillance decision support system integrating PPC risk prediction with structured nursing action recommendations. **Methods**: In this retrospective cohort study, electronic medical record (EMR) data from approximately 6900 adult patients who underwent abdominal surgery at a single institution between January 2015 and September 2023 were analyzed. The study protocol was approved by the Institutional Review Board, and the requirement for informed consent was waived because of the retrospective study design. PPC risk was predicted using a tabular multilayer perceptron (MLP) encoder with SHapley Additive exPlanations (SHAP)-based feature weighting and a random forest classification head optimized via Optuna. Class imbalance was addressed using weighted sampling, class weighting in BCE(Binary Cross Entropy) With Logits Loss, and decision-threshold optimization. For clinical decision support, a large language model generated structured nursing surveillance recommendations in an action–evidence–rationale JSON format and was aligned through supervised fine-tuning (SFT) using human-evaluated cases. **Results**: The prediction model achieved an AUROC of 0.810, with an accuracy of 0.811, precision of 0.547, and recall of 0.545. In expert evaluation, the SFT-aligned model improved recommendation quality, reducing incorrect nursing actions from 19.3% to 8.0%. **Conclusions**: The proposed system demonstrates the feasibility of an end-to-end nursing surveillance decision support framework linking PPC risk prediction with structured clinical recommendations. The findings suggest its potential to support more accurate risk prediction and more actionable nursing surveillance for patients undergoing abdominal surgery.

## 1. Introduction

Postoperative complications remain a major threat to surgical quality and patient safety, particularly in patients undergoing major abdominal surgery [1,2,3]. Among postoperative complications, PPCs are particularly important after abdominal surgery because they range from relatively mild conditions, such as atelectasis and pleural effusion, to severe events including pneumonia, reintubation, and respiratory failure [4,5]. Recent studies have reported PPC incidence rates of 22.5% in emergency abdominal surgery and 8.7% in elderly patients undergoing major abdominal surgery, suggesting that the incidence varies according to surgical setting, patient characteristics, and diagnostic criteria [4,5]. PPCs are particularly common and clinically significant in older adults and in patients with underlying pulmonary disease or impaired lung function [5]. Beyond their frequency, PPCs are associated with a prolonged length of stay, increased medical costs, and higher short-term mortality [5,6]. For example, in elderly patients undergoing abdominal surgery, the median hospital stay was reported to be 30 days in the PPC group versus 18 days in the non-PPC group, indicating a substantial adverse effect on postoperative recovery [5].

From a nursing perspective, the early identification of PPC risk is closely related to nursing surveillance, which has been defined as a purposeful and ongoing process of collecting, interpreting, and synthesizing patient data to support clinical decision-making [7,8]. However, nursing surveillance should not be reduced to routine observation or documentation alone. Rather, it is a core professional nursing activity that requires nurses to detect subtle physiologic changes, interpret their clinical significance, and initiate timely responses. This process can be theoretically supported by Tanner’s Clinical Judgment Model, which conceptualizes nursing practice as noticing, interpreting, responding, and reflecting [9]. In postoperative care, this means that nurses must recognize early respiratory warning signs, determine whether they indicate clinically meaningful deterioration, and translate this judgment into appropriate monitoring or intervention. The AACN Synergy Model further reinforces this perspective by emphasizing that optimal patient outcomes depend on the alignment between patient needs and nursing competencies, thereby underscoring surveillance as a central component of safe postoperative care [10]. Nevertheless, effective nursing surveillance is increasingly challenged in real-world practice by documentation burden and excessive workload, making the consistent recognition of subtle deterioration difficult even for experienced nurses [11].

To address postoperative risk, various prediction tools have been developed, including conventional scoring systems such as ARISCAT and more recent machine learning models based on perioperative or electronic medical record (EMR) data [12,13,14,15]. However, the limitations of existing approaches remain substantial. First, many conventional PPC prediction models were primarily developed for preoperative risk stratification and provide only static risk estimates, limiting their usefulness for ongoing bedside surveillance after surgery [14,15]. Second, although recent artificial intelligence (AI)-based models have improved predictive performance, most function mainly as classification or alerting tools and provide little guidance regarding what nurses should monitor or how they should respond once high risk is identified [12,13]. Third, prior models often rely heavily on structured variables while underutilizing clinically meaningful surveillance signals documented in nursing records, even though early respiratory deterioration is often first recognized through bedside nursing assessment. In addition, recent external validation studies have shown that many PPC prognostic models demonstrate limited generalizability and inadequate calibration across settings, raising concerns regarding their clinical robustness [16]. Taken together, current approaches do not adequately bridge the gap between risk prediction and nurse-led action.

Accordingly, the unmet need is not simply for a more accurate classifier, but for a nursing-centered decision support system that connects prediction, surveillance, and intervention. In response to this gap, this study proposes a holistic nursing surveillance decision support system for PPCs after abdominal surgery by integrating a deep learning-based risk prediction model using EMR data with a large language model (LLM)-based recommendation module [10,11]. Using data from approximately 6900 patients, the proposed framework incorporates surgical characteristics, comorbidities, physiologic indicators, and symptom-related variables derived from nursing documentation. Unlike prior models that mainly provide risk alerts, the proposed system is designed to support clinical nursing judgment by translating predicted risk and patient status into predefined, structured nursing surveillance recommendations in an action–evidence–rationale format.

The main contributions of this study are as follows:

(1) We developed a multi-source PPC prediction framework integrating structured EMR features and symptom-related indicators derived from nursing records.
(2) We proposed a hybrid architecture combining a Tabular MLP encoder with a Random Forest head, together with SHAP-based weighting and imbalance-aware learning strategies.
(3) We developed an LLM-based decision support module that generates predefined nursing surveillance actions in a structured action–evidence–rationale JSON format based on predicted PPC risk and patient condition, and refined this module through supervised fine-tuning (SFT) with expert-informed evaluation.

## 2. Materials and Methods

### 2.1. Data Collection

This retrospective cohort study protocol was approved by the Institutional Review Board (IRB) of Changwon National University. The requirement for informed consent was waived due to the retrospective nature of the study. Electronic Medical Record (EMR) data were collected from the Clinical Data Warehouse of Samsung Changwon Hospital between January 2015 and September 2023. The hospital is located in a non-metropolitan area.

### 2.2. Patients and Postoperative Pulmonary Complications

This study included approximately 6900 adult patients aged 19 years or older who underwent abdominal surgery. The dataset used in this study consisted of three main components, as described below:

- Patient characteristics: Basic demographic and clinical information, such as age, sex, body mass index (BMI), and the presence of underlying comorbidities.
- Surgery- and anesthesia-related information: Details, such as the surgical site, surgical approach, and anesthesia time.
- Nursing notes and vital signs: Respiratory rate, oxygen saturation (SpO_2_), body temperature, heart rate, pain score, and characteristics of cough and sputum.

To address the high risk of overfitting associated with including numerous variables in a single-center dataset, a structured *a priori* variable selection and reduction strategy was implemented. Initially, potential predictive variables were selected based on PPC predictors identified in previous studies, followed by a rigorous review process by nursing domain experts. During the subsequent data preprocessing phase, a strict variable reduction process was applied; specifically, variables with a high proportion of missing values or a lack of clinical consistency were explicitly excluded. The current list of variables utilized in this study is the direct result of this systematic selection and reduction process.

The exclusion criteria for this study were as follows: patients who underwent concurrent surgeries on non-abdominal sites; patients who underwent two or more surgeries during the same hospitalization; patients who did not receive general anesthesia; patients with missing diagnostic codes; and cases where preoperative and postoperative records could not be distinguished. Patients meeting any of these criteria were excluded. Patients with PPCs were identified based on radiological findings from imaging tests (chest CT, chest X-ray) suggestive of pneumonia, atelectasis, pleural effusion, pulmonary edema, pneumothorax, or related conditions. Findings marked as “rule out (R/O)” or “suspicious” were also included in this category. However, these cases were not intended to represent definitive PPC diagnoses but rather to define a surveillance population at risk of pulmonary complications. This approach was adopted to capture clinically relevant situations requiring monitoring, rather than restricting the cohort to only confirmed cases. Therefore, the outcome definition reflects a pragmatic identification of patients requiring clinical attention, and the potential for bias arising from this inclusive definition should be considered when interpreting the results.

### 2.3. Dataset

Table 1 summarizes the variables used in this study, including feature names, data types, and definitions. The input features integrated structured EMR data and unstructured nursing documentation across four domains: patient characteristics, surgery- and anesthesia-related information, vital signs, and nursing-note-derived indicators. The final feature set included more than 40 variables, comprising numeric, categorical, and Boolean features. The temporal scope of data collection was defined as the postoperative period from immediately after surgery to hospital discharge, and all PPC-related predictors and outcomes were derived within this surveillance window.

Only one variable initially contained missing values. Because this variable showed the lowest feature importance and limited clinical relevance based on expert review, it was excluded from the dataset. Consequently, the final analytic dataset contained no missing values, and no imputation procedure was required.

Baseline characteristics stratified by PPC status are presented in Supplementary Table S1. In the overall cohort (*N* = 6263), gallbladder surgery and laparoscopic procedures accounted for the largest proportions of cases. However, the PPC group showed a clearly different clinical profile from the non-PPC group. Patients with PPCs were older, more often male, and more likely to undergo open surgery with longer anesthesia duration. They were also more frequently represented in higher-risk surgical and diagnostic categories, including stomach and liver surgery and hepatobiliary–pancreatic malignancy-related diagnoses. These findings suggest that PPC occurrence was associated with greater surgical invasiveness and a higher baseline disease burden.

The PPC group also demonstrated less favorable baseline health conditions, including more complex comorbidity patterns, smoking history, reduced functional status, L-tube insertion, emergency room admission, and obesity. In addition, postoperative physiologic instability was consistently more prominent in the PPC group, with higher frequencies of abnormal blood pressure, body temperature, respiratory rate, pulse rate, SpO_2_, and pain scores. Nursing-note-derived symptom indicators, including systemic infection symptoms, bronchial secretion symptoms, and respiratory symptoms, were also more common in the PPC group.

Overall, Supplementary Table S1 shows that PPC risk was associated with the combined influence of baseline vulnerability, operative burden, and evolving postoperative physiologic and symptom-based instability. These findings support the inclusion of both structured physiologic variables and nursing-documentation-derived indicators in the predictive framework and reinforce the clinical relevance of a nursing-centered surveillance approach for PPC identification.

### 2.4. Problem Definition

In this study, we formulated (1) a binary classification problem to predict whether a surgical patient will develop PPCs, and (2) a multi-label/multi-class problem to recommend appropriate nursing interventions, selected from a predefined set of 12 items, based on the prediction results and patient-specific characteristics. The primary objective of this study was to use patient data to predict the occurrence of PPCs. The secondary objective was to identify and propose interventions needed for the patient from a predefined set of 12 nursing interventions, based on the predicted presence or absence of pulmonary complications and the patient’s clinical status. In this secondary intervention recommendation stage, we utilized a large language model (LLM) that takes the prediction model outputs and patient data as inputs and generates a structured nursing surveillance decision-support format that includes evidence and rationale.

### 2.5. Model Pipeline

**Figure 1:**
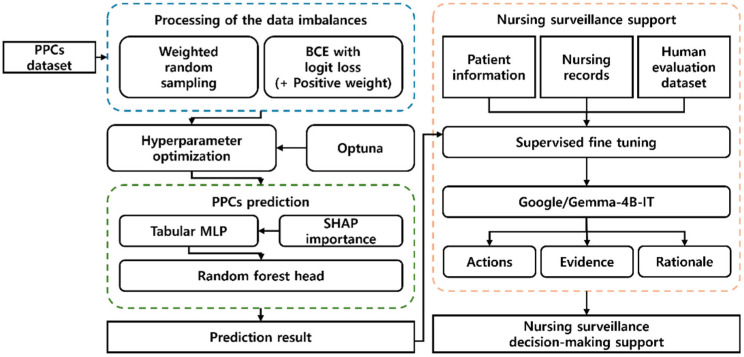
Proposed Model Pipeline. PPCs = postoperative pulmonary complications, BCE = binary cross entropy, MLP = multilayer perceptron, and IT = instruction tuned.

Figure 1 illustrates the overall model pipeline, which comprises three major stages based on the PPCs dataset. First, in the data imbalance handling stage, the class imbalance problem is addressed while preserving the original data without discarding samples by using weighted random sampling and a loss function that applies a positive-class weight (pos_weight) to BCE With Logits Loss during training. Next, the hyperparameters were automatically searched and tuned using the Optuna module, and the pipeline proceeded to the second stage, the postoperative pulmonary complication prediction model. In this study, predictions were performed using patient data collected during the postoperative period, from immediately after surgery to hospital discharge. The model was designed to dynamically assess the risk of PPCs during this period, reflecting changes in patient condition over time rather than relying on a single fixed time point.

In the pulmonary complication modeling stage, a deep learning model, Tabular MLP, receives multivariate inputs, and model training is performed by applying SHapley Additive exPlanations (SHAP)-importance-based weighting to the input variables. The multivariate features were transformed into latent representations using a deep neural network encoder, which were then used by a RF classifier to produce the final prediction and output the results.

Finally, in the nursing surveillance decision-support format generation stage, we fine-tune an LLM by combining the prediction results from the previous stage, patient information data, and a human evaluation dataset in which nursing surveillance decisions for patient information are labeled. The SFT-trained model then generates and provides structured nursing surveillance decision support output in an action, evidence, and rationale format.

### 2.6. Postoperative Pulmonary Complication Prediction Model

The PPC prediction model inputs raw data into an MLP-based encoder after preprocessing (e.g., normalization) and a feature-importance-based weighting step. The encoder directly receives normalized continuous inputs, while categorical inputs are transformed into embedding vectors for use. It learns complex patterns in the input through multiple fully connected hidden layers. The node values immediately before the final output layer of the encoder were regarded as the latent representations for each patient sample. The resulting representation vectors were then used as inputs to a random forest (RF) classification head to perform a binary classification of whether pulmonary complications occurred. The MLP encoder and RF classifier were trained in separate stages: first, the MLP encoder was pre-trained, and then the embedding vectors for each sample were extracted from the encoder and used as training inputs for the RF model.

From a clinical perspective, this hybrid architecture was deliberately selected to address the inherent limitations of deploying pure deep learning models in healthcare settings. While models combining deep learning and traditional machine learning are already widely used, pure neural networks are notoriously opaque (the “black-box” problem), which heavily hinders clinical trust. By integrating an MLP, which efficiently captures complex, non-linear relationships among heterogeneous patient variables, with an RF classifier—a traditional algorithm renowned for its structural transparency and stable generalization—this pipeline bridges the gap between high predictive accuracy and clinical interpretability. Through this design, we aimed to achieve strong performance even with small sample sizes while ensuring that the model’s decision-making process is transparent and reliable enough for clinicians to trust in real-world practice. The RF classifier outputs a probability value between zero and one; if this value exceeds a predefined decision threshold, the case is predicted to be positive (PPC occurrence).

#### 2.6.1. Handling Class Imbalance

To address the class-imbalance problem, in which the number of patients who develop pulmonary complications is much smaller than the total number of surgical patients, we applied several weight-based techniques. First, during training of the deep learning encoder, we used BCE With Logits Loss with the positive weight option to impose a larger penalty for errors on the positive class (PPC occurrence). The positive weight value is typically set to the ratio of negative to positive samples; in this study, it was set to approximately 8:2, according to the distribution in the training data. Second, we used a weighted random sampler for batch sampling to ensure that each mini-batch included a minimum proportion of positive cases. Specifically, while iterating over the entire dataset within an epoch, we assigned weights so that positive samples could be repeatedly selected, thereby increasing the effective proportion of positive samples in each batch. Third, when training the RF classifier, we used scikit-learn’s class_weight = “balanced” option so that the tree-splitting criteria would place greater weight on positive-class errors. Fourth, for the final prediction, we used an adjusted decision threshold rather than 0.5 to determine positive and negative outcomes. The threshold was determined using the validation data at the point that optimized the balance between precision and recall. Because imbalanced data often require lowering the threshold below 0.5 to improve recall, we selected the probability value that maximized the F1-like score. Finally, we conducted experiments using Focal Loss [17].

Focal Loss is a loss function that encourages the model to focus on learning the minority class under imbalance by amplifying the loss for hard examples while down weighting the contribution of easy examples. In this study, we performed an Optuna-based hyperparameter search that included the hyperparameters of the Focal Loss, thereby automatically determining whether to use the Focal Loss and its optimal values. Consequently, the final model adopted weighted BCE loss instead of Focal Loss, likely because applying class weights alone was sufficient to achieve favorable recall on this dataset. Notably, we did not use synthetic oversampling techniques, such as SMOTE. Although SMOTE can artificially increase the number of minority class samples, it may introduce noisy samples or increase the risk of overfitting in small medical datasets; therefore, we replaced it with a cost-sensitive learning approach, which has been recommended in recent studies.

#### 2.6.2. Hyperparameter Optimization

The key hyperparameters of the model were automatically searched using the Optuna [18] module. Optuna is a library that efficiently explores parameter combinations using an advanced Bayesian optimization algorithm and enables easy optimization through user-defined objective functions. In this study, we conducted an Optuna search over approximately 10 items, including encoder and classifier architectures, learning rates, regularization terms, and feature-weighting parameters. The target metric for optimization was set to the receiver operating characteristic area under the curve (ROC-AUC) of the validation dataset, and a search was performed to maximize the mean AUC using five-fold cross-validation.

The specific tuning parameters are as follows: batch size, initial learning rate, MLP dropout ratio, MLP hidden-layer sizes, feature embedding dimension, number of trees, maximum depth of the RF, SHAP weighting parameters, loss function type (BCE vs. focal), and focal loss parameters. For each parameter set suggested by Optuna, we trained the encoder + RF model and evaluated the validation AUC; the process converged after approximately 500 trials. The final selected hyperparameters included a batch size of 256, a learning rate of 2.16 × 10^−3^, a dropout rate of 0.43, an MLP hidden1 size of 256, an MLP hidden2 size of 64, 200 RF trees (max_depth = 10), a SHAP weight scaling coefficient of 0.27, and an exponent of 0.82. Through this automated tuning process, we could obtain an optimal combination more quickly than by manual tuning, thereby further improving the model performance.

#### 2.6.3. SHAP-Based Feature Importance Extraction

To enhance model interpretability and to guide learning toward clinically relevant predictors, we performed a feature importance analysis using SHAP values. SHAP [19], proposed by Lundberg and Lee, estimates the contribution of each input variable to a model prediction based on Shapley values. In this study, SHAP values were calculated for individual patients and subsequently aggregated to support both global and local interpretation of PPC risk.

**Figure 2:**
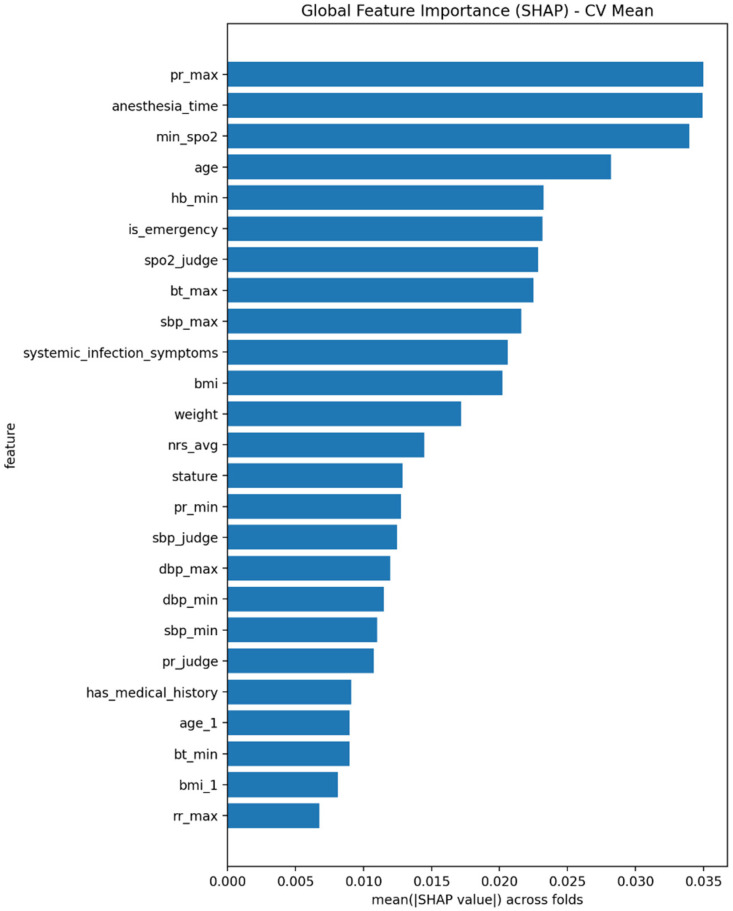
Top Features by SHAP Importance.

Figure 2 presents the global importance of the top variables based on the mean absolute SHAP values averaged across cross-validation folds. The highest-ranked variables were pr_max, anesthesia_time, min_spo_2_, and age, followed by hb_min, is_emergency, spo_2__judge, bt_max, sbp_max, and systemic_infection_symptoms. Overall, this pattern suggests that PPC risk was shaped by the combined influence of perioperative burden, postoperative respiratory instability, hemodynamic stress, inflammatory response, and baseline patient vulnerability, rather than by a single dominant predictor. The prominence of abnormal vital-sign flags and nursing-note-derived symptom indicators further suggests that the model captured not only raw physiologic measurements but also clinically interpreted surveillance signals that are meaningful in postoperative nursing practice.

**Figure 3:**
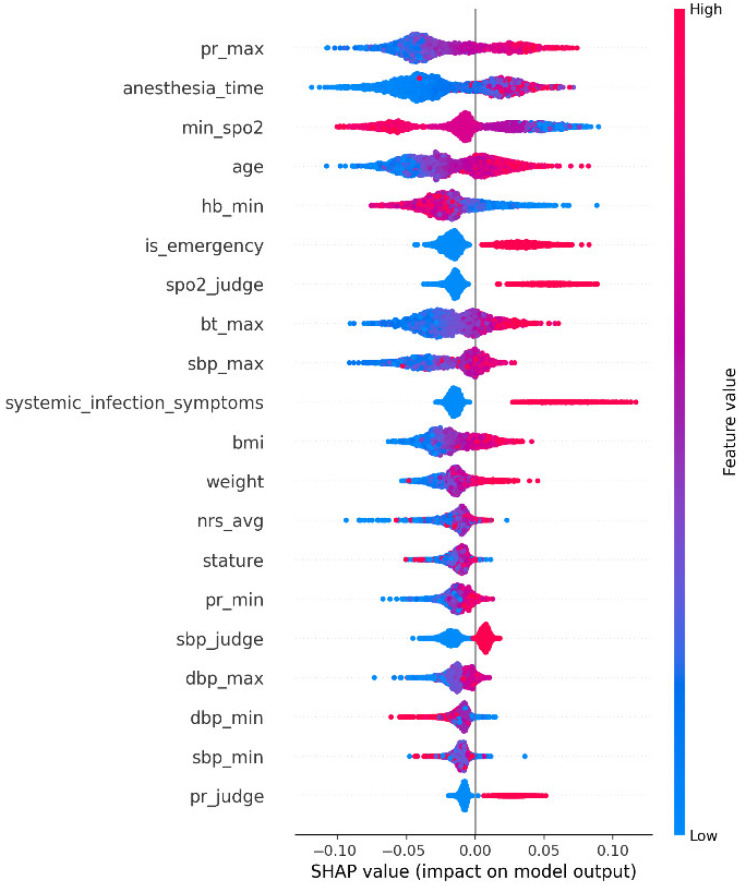
SHAP Analytical Interpretation Table.

Figure 3 provides complementary information by illustrating how different feature values influenced model output. In the SHAP summary plot, each point represents an individual patient, the horizontal axis indicates the magnitude and direction of the SHAP contribution, and the color reflects the feature value from low (blue) to high (red). Positive SHAP values indicate contributions toward higher predicted PPC risk, whereas negative SHAP values indicate contributions toward lower risk. Several clinically coherent patterns were observed. Higher PR_max, longer anesthesia_time, older age, and higher bt_max were generally associated with positive SHAP values, indicating increased PPC risk. In contrast, lower min_SpO_2_ and lower Hb_min tended to shift predictions toward higher risk, suggesting that oxygen desaturation and reduced hemoglobin were important adverse signals. Binary indicators such as SpO_2__judge, SBP_judge, and PR_judge also demonstrated directional separation, supporting the interpretation that clinically flagged abnormalities contributed consistently to increased model-predicted risk. In addition, systemic_infection_symptoms showed a tendency toward positive SHAP contributions, implying that postoperative inflammatory or infectious manifestations may also be relevant to PPC development.

These SHAP findings provide clinically meaningful implications beyond technical interpretability. First, the high importance of min_SpO_2_, SpO_2__judge, and PR_max suggests that early respiratory compromise and cardiopulmonary instability are central warning signs for PPC development. This finding supports close monitoring of oxygen saturation, respiratory status, and pulse rate during the postoperative period. Second, the contribution of anesthesia_time and is_emergency indicates that procedural burden and surgical urgency should be considered when determining the intensity of postoperative surveillance, particularly in patients undergoing prolonged or emergency surgery. Third, the importance of age and Hb_min suggests that baseline physiologic vulnerability and reduced reserve remain relevant even before overt respiratory deterioration becomes clinically apparent. Finally, the contribution of systemic_infection_symptoms and abnormal vital-sign indicators suggests that PPC surveillance should not be restricted to isolated respiratory variables alone but should instead be interpreted within a broader context of postoperative deterioration. Taken together, these findings indicate that SHAP results are valuable not only for explaining model output but also for translating PPC prediction into concrete bedside surveillance priorities for nurses.

SHAP also enabled local interpretation at the individual patient level. For example, if a 75-year-old patient was predicted to be at high risk for PPC, the patient’s SHAP profile might indicate that high maximum pulse rate, older age, prolonged anesthesia time, low postoperative oxygen saturation, and low hemoglobin were the major contributors. Such case-level explanations may help nurses and clinicians understand why a patient was classified as high risk and may facilitate earlier escalation, closer respiratory assessment, and more targeted surveillance.

In addition to post hoc interpretation, we incorporated SHAP-derived feature importance into model training through a feature-reweighting strategy. To estimate variable importance at an early stage, we first trained a simplified RF model, computed SHAP values on the training dataset, and used the mean SHAP value of each variable as an importance indicator. After normalization, the feature weight was defined as follows:

$$w_{i}=1+\;\lambda \;\cdot {\left({imp}_{i}\right)}^{\gamma }$$

where ${imp}_{i}$ denotes the normalized importance of feature i, and λ and γ are hyperparameters controlling scaling strength and nonlinearity, respectively. Using this formulation, variables with higher estimated importance received larger scaling factors at the encoder input, whereas lower-ranked variables received relatively smaller weights. This design was intended to improve sensitivity to clinically important predictors while preserving the full feature set.

However, this weighting strategy should be interpreted with caution. Because the weights are derived from feature importance estimated by a preliminary RF model, the approach may inherit bias if the initial importance estimates are unstable, sample-specific, or dependent on institutional data characteristics. In particular, excessive pre-scaling may overemphasize variables that appear important in the development dataset while reducing sensitivity to weaker but still clinically meaningful predictors. For this reason, the proposed weighting approach should not be regarded as a replacement for standard feature selection methods. Rather than eliminating variables, it preserves all inputs and modulates their relative emphasis during representation learning. In the present study, SHAP-based weighting should therefore be understood as an interpretable feature reweighting strategy designed to guide representation learning, while acknowledging the possibility of bias and the need for careful interpretation.

### 2.7. Nursing Surveillance Activiy Recommendation Model

The nursing surveillance activity recommendation model is based on an LLM that generates appropriate nursing surveillance activities by synthesizing the previously predicted risk of PPCs and the patient’s clinical status.

Specifically, to recommend surveillance action, we provided the LLM with a prompt that included the patient’s key clinical information. The prompt contains patient information, nursing surveillance records, and whether a pulmonary complication has occurred. It also includes an instruction regarding the output format, such as a command to “provide nursing surveillance action in JSON format.” The LLM interprets this prompt and generates an answer according to a predefined JSON template in a structure such as {“action”: […], “evidence”: “…”, “rationale”: “…”}.

Here, action refers to the list of recommended nursing surveillance activities based on patient information, evidence refers to the patient information features that serve as the basis for recommending action(s), and rationale provides an interpretation explaining why the surveillance activities are needed. For example, for a high-risk patient, the action field may include items such as “Monitor oxygen saturation (SpO_2_)” and “Verify intervals for vital signs and SpO_2_ monitoring,” while the evidence field may include a feature such as “Oxygen saturation: 90% (threshold < 95%),” and the rationale field may include an explanation such as “Because the patient’s SpO_2_ level is below the acceptable threshold, immediate monitoring and intervention are required.” Thus, the design leverages the LLM’s free-form generation capability while requiring a JSON format to ensure structured outputs. The JSON format is advantageous for integration with EMR systems and for managing multiple recommended surveillance activities in a database.

#### 2.7.1. Types of Nursing Surveillance Action

In this study, we organized the surveillance activities for the prevention and management of PPCs into 12 nursing surveillance actions. These actions were derived from a review of relevant literature, clinical guidelines, and expert consultation. The 12 actions presented in Table 2 represent a comprehensive framework of nursing surveillance activities required for effective pulmonary complication monitoring. Although each action is conceptually distinct, they may be interrelated and applied in combination depending on the patient’s condition and risk factors. For example, in patients at high risk, multiple surveillance actions may be implemented simultaneously from different clinical perspectives. In contrast, for patients at low risk, only basic actions, such as breathing exercises and encouragement of early ambulation, may be sufficient. This flexible application reflects the practical characteristics of nursing surveillance, in which actions are tailored to patient status rather than applied in isolation.

#### 2.7.2. Nursing Surveillance Action Recommendation Model

**Figure 4:**
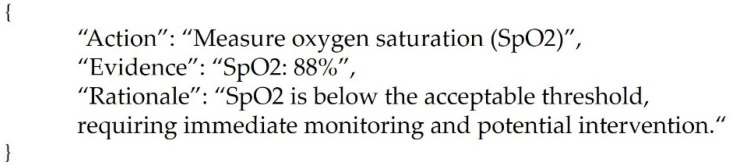
Nursing Surveillance Decision Support Format.

For the LLM used in the nursing surveillance activity recommendation model, Google Gemma-3-4B-IT (Google DeepMind, Mountain View, CA, USA) was employed. To obtain high-quality outputs, we partially referred to the structured prompting technique proposed by Cao et al. [20] when designing the prompts. First, patient status inputs were provided as itemized bullet points (e.g., Age: 82 years; Sex: Female; Surgical site: gallbladder). We then added role and format instructions such as “You are a highly precise nursing surveillance support system. This patient was at a high risk of developing pulmonary complications. Provide a list of nursing interventions for the patient below and output the evidence and theoretical explanations in JSON format. Following these instructions, Gemma-3 generated relatively consistent JSON outputs. In cases where formatting errors occurred or the model produced unnecessarily verbose responses, we improved the output by adjusting the prompt. An example of a finalized LLM output is shown in Figure 4. For more details on prompts, please refer to Appendix A.

Among patients with confirmed pulmonary complications, we randomly selected 100 patients and developed a nursing surveillance decision-support format. We constructed this dataset, conducted a human evaluation, and performed SFT on the LLM using correctly generated cases from the evaluation results. This was conducted to align the model by training it on human-evaluated outputs, thereby avoiding the production of incorrect results.

Thus, the LLM proposed concrete surveillance activities tailored to each patient’s individual situation and described the supporting evidence. The generated surveillance activities can be executed directly by nurses or used as references, and providing evidence and explanations can yield educational benefits. Although the integration of the prediction model and LLM module is currently implemented separately in a prototype form, the plan is to ultimately build a single pipeline linked to the hospital information system. In this study, as a development stage, we conducted a small-scale evaluation of the integrated prediction–intervention performance.

## 3. Results

### 3.1. Experimental Setup

In the experimental setup of this study, we used EMR from surgical patients collected at SC Hospital. To predict PPCs, we developed a Tabular MLP model combined with an RF head as the classification layer. Model training was implemented in Python(version 3.12.13) using the PyTorch(version 2.10.0+cu128) and scikit-learn libraries(version 1.6.1). The MLP encoder was trained using PyTorch for up to 50 epochs with early stopping. Adam was used as the optimizer, and to reduce instability in the early phase of training, we applied a warm-up strategy for approximately five epochs, starting with a low learning rate and gradually increasing it. The RF classifier was trained separately using Scikit-learn’s implementation, taking the embedding vectors extracted from the pre-trained encoder as input. The training and validation data for the hyperparameter search were randomly split from the full dataset at an 80:20 ratio. The final performance evaluation was conducted using a holdout test set (20%) that was not used during training. The Optuna search and SHAP computations were partially parallelized to reduce the computational cost. We used Google’s Gemma-3-4B-IT model for nursing surveillance activity recommendations. For comparative experiments evaluating PPC prediction performance, we included machine learning–based models, namely Random Forest (RF), Logistic Regression (LR), XGBoost (XGB), and LightGBM (LGBM), as well as deep learning–based models, including Tabular MLP, FT-Transformer, and TabNet. The proposed model and deep learning baseline models were trained in the same computational environment (Google Colab A100), with early stopping applied during training for up to 50 epochs where applicable.

Model performance was evaluated using seven metrics: precision, recall, accuracy, F1-macro, ROC-AUC, PR-AUC, and specificity. F1-macro was used to reflect balanced performance across classes under class imbalance, while PR-AUC was additionally included to provide a more informative assessment of positive-class detection in the imbalanced setting.

### 3.2. Experimental Results

The quantitative results of the experiments are summarized in Table 3. To establish a robust benchmark and provide direct comparisons with methods commonly used in clinical prediction studies, we included conventional machine learning models, such as Logistic Regression and Random Forest, as well as advanced tree-based and deep learning architectures. Overall, the proposed model showed the most favorable point estimates across several key evaluation metrics compared with both machine learning (ML) and deep learning (DL) baselines.

Because the primary clinical objective of this study was to identify patients at risk of postoperative pulmonary complications (PPCs), minimizing false negatives was considered important to support patient safety and timely intervention. Accordingly, recall, which is equivalent to sensitivity in binary classification, was regarded as a clinically important metric because it reflects the ability to correctly identify positive cases. At the same time, specificity was also evaluated to determine how well the model avoided incorrectly classifying low-risk patients as positive. Because excessive false positives may increase unnecessary alerts and clinical workload, precision was additionally considered. To reflect the balance between precision and recall, F1-macro was used as a key summary metric, particularly under class imbalance. For the proposed model, the final classification threshold was set to 0.517 on the basis of validation data rather than using the default threshold of 0.5, in order to achieve a more appropriate balance between sensitivity and specificity.

The proposed model achieved the highest point estimates for ROC-AUC (0.810), accuracy (0.810), F1-macro (0.712), PR-AUC (0.531), and specificity (0.879). The corresponding 95% confidence intervals were 0.782–0.839 for ROC-AUC, 0.789–0.832 for accuracy, 0.681–0.742 for F1-macro, 0.470–0.596 for PR-AUC, and 0.859–0.900 for specificity. In comparison, among the ML baselines, Random Forest showed the highest ROC-AUC (0.785; 95% CI, 0.771–0.800), whereas among the DL baselines, FT-Transformer showed the highest ROC-AUC (0.783; 95% CI, 0.769–0.797). TabNet demonstrated the lowest overall performance, with an ROC-AUC of 0.741 (95% CI, 0.727–0.756), an F1-macro of 0.657 (95% CI, 0.644–0.671), and a PR-AUC of 0.430 (95% CI, 0.404–0.458).

In terms of the precision–recall trade-off, the proposed model achieved a recall (sensitivity) of 0.546 (95% CI, 0.489–0.606), which was lower than that of FT-Transformer (0.615; 95% CI, 0.586–0.643) and Logistic Regression (0.578; 95% CI, 0.541–0.595). However, the proposed model achieved substantially higher precision, with a value of 0.544 (95% CI, 0.484–0.608), compared with FT-Transformer (0.445; 95% CI, 0.423–0.470) and Logistic Regression (0.473; 95% CI, 0.432–0.482), indicating fewer false-positive predictions. In addition, the proposed model showed the highest specificity, 0.879 (95% CI, 0.859–0.900), compared with 0.798 (95% CI, 0.786–0.810) for FT-Transformer and 0.825 (95% CI, 0.810–0.833) for Logistic Regression. These findings indicate that the proposed model achieved a more balanced operating point, maintaining competitive sensitivity while reducing excessive false positives that could otherwise increase clinical burden.

The proposed model also achieved the highest F1-macro score, 0.712 (95% CI, 0.681–0.742), whereas the baseline models ranged from 0.657 to 0.682. Likewise, it achieved the highest PR-AUC, 0.531 (95% CI, 0.470–0.596), suggesting improved overall performance in identifying positive PPC cases under class imbalance. When interpreted together with the confidence intervals, these results suggest that the proposed model showed favorable and stable performance across multiple complementary metrics. Overall, the findings indicate that the proposed model provides a clinically meaningful balance between sensitivity and specificity while maintaining strong discriminative ability and precision.

### 3.3. Ablation Study

Table 4 presents the ablation study results, showing how each methodological component contributed to the performance of the proposed model. To validate the effectiveness of our framework, we compared the full model (“Ours”) with three reduced variants: a model without SHAP-based feature importance weighting, a model without imbalance handling, and the baseline model.

First, removing SHAP-based feature importance weighting led to a consistent decline across all evaluation metrics. Compared with the full model, the variant without SHAP importance showed lower Precision (0.508 vs. 0.544), Recall (0.515 vs. 0.546), Accuracy (0.795 vs. 0.810), F1-macro (0.691 vs. 0.712), ROC-AUC (0.791 vs. 0.810), PR-AUC (0.470 vs. 0.530), and Specificity (0.868 vs. 0.879). These findings suggest that SHAP-based weighting helped the model place greater emphasis on clinically informative variables, thereby improving both discrimination and overall classification performance.

Second, removing the imbalance handling strategy also resulted in lower overall performance. The model without imbalance handling achieved Precision of 0.506, Recall of 0.508, Accuracy of 0.794, F1-macro of 0.688, ROC-AUC of 0.794, PR-AUC of 0.509, and Specificity of 0.869, all of which were lower than those of the full model. This result indicates that addressing class imbalance contributed meaningfully to balanced predictive performance, particularly in terms of Recall, F1-macro, and PR-AUC.

Finally, the baseline model showed the lowest overall performance in most metrics, with Precision of 0.494, Recall of 0.496, Accuracy of 0.789, F1-macro of 0.681, ROC-AUC of 0.800, PR-AUC of 0.498, and Specificity of 0.866. In contrast, the full model achieved the best overall results, including the highest Accuracy (0.810), F1-macro (0.712), ROC-AUC (0.810), PR-AUC (0.530), and Specificity (0.879). These results support that the combined application of SHAP-based feature importance weighting and imbalance handling produced the most robust predictive performance among all compared configurations.

### 3.4. Human Evaluation

In the human evaluation stage, we assessed whether the surveillance activity recommendation system provided appropriate interventions, supporting evidence, and interpretations through expert reviews. The evaluation was conducted by having nursing-domain experts score the system-generated responses. We randomly selected recommendations for 100 patients from the full set of generated outputs for the assessment. In the scoring scheme, responses were given five points when the model provided the correct recommended actions, evidence, and interpretations, and one point was assigned when the generated response was incorrect. Because multiple actions can be suggested for a single patient, we averaged the scores of the actions presented for each patient and summarized the results into five scores, as shown in Table 5.

The base model is without an SFT. The model produces 389 recommended surveillance activities for 100 patients. According to the human evaluation results, 314 actions received 5 points, whereas 75 actions were judged inappropriate and received 1 point. The proposed method is based on an SFT-trained model. It generated 301 recommended surveillance activities, assigning 5 points to 277 actions and 1 point to 24. While the model without SFT produced incorrect outputs at a rate of 19.3% for all actions, the SFT-trained model reduced this rate to 8%, indicating an improvement of approximately 11 percentage points. In the comparative results shown in Table 5, 52% of the cases for the base model received a score of 5, and a relatively large proportion of patients received scores of 3 and 2. In contrast, in our study, 91% of the 100 patients were concentrated in the five- and four-point categories. These results indicate that the SFT-trained model exhibits fewer hallucinations and provides a more accurate nursing surveillance decision support format when generating responses.

### 3.5. Case Study

Table 6 compares the example outputs of the proposed model (Ours) and the baseline model. This table presents the nursing surveillance decision-support formats generated by the two models for the same patient. The nursing actions highlighted in red indicate actions recommended by the baseline model and illustrate cases in which the evidence and interpretation were incorrectly matched. In contrast, the nursing actions highlighted in blue indicate actions recommended by the proposed model and demonstrate cases in which the evidence and interpretation were appropriately matched with the action.

When analyzing the case, the nursing surveillance decision-support format generated by our model recommended actions such as monitoring oxygen saturation and verifying the intervals for vital sign monitoring. In this case, hypertension was presented as the supporting evidence, and the action, evidence, and rationale were systematically linked, showing that the model proposed actions based on appropriate evidence. In contrast, the baseline model suggested an action related to respiratory rate and breathing pattern while presenting systolic blood pressure as the evidence feature, resulting in a mismatch between the action and the evidence. In addition, it generated an output suggesting excessive intervention even though the patient’s body temperature was within the normal range.

Based on these results, we confirmed that SFT guided by human evaluation helps prevent the model from proposing excessive actions and enables more accurate decision support by more precisely aligning actions with their supporting evidence when generating the nursing surveillance decision-support format.

## 4. Discussion

This study proposes a nursing-centered clinical decision support system (CDSS) for postoperative pulmonary complications (PPCs) in abdominal surgery patients by integrating prediction, explanation, and intervention. Using single-center retrospective data, we demonstrated the feasibility of a framework that translates PPC risk estimates into structured nursing surveillance recommendations. The significance of this system lies not merely in estimating risk, but in supporting nursing surveillance, clinical judgment, and timely response in postoperative care.

These findings should be interpreted cautiously. Although the model showed acceptable discrimination, predictive performance alone does not ensure clinical usefulness when external validity, calibration, and implementation context remain unestablished [21,22]. Therefore, the present results should be regarded as evidence of feasibility within the source institution rather than proof of broad clinical safety or general applicability. Transferability is a particularly important concern. Because the model was developed retrospectively at a single institution, its performance may reflect local patient mix, perioperative practice patterns, documentation behavior, and institutional PPC labeling processes. In addition, nursing-note-derived indicators and institution-specific EMR variables may not be directly portable to hospitals with different EMR structures, documentation templates, and surveillance workflows. Accordingly, broader implementation would likely require local feature remapping, recalibration, threshold adjustment, and prospective validation before deployment [22,23].

An additional methodological consideration concerns the outcome definition, specifically the potential risk of bias introduced by including “suspected” or “rule-out” conditions. In this study, the outcome was explicitly designed not as a strict classification of a definitive PPC diagnosis, but as a pragmatic identification of an “at-risk” postoperative surveillance population. While this inclusive approach reflects the clinical reality that patients with suspected pulmonary complications require closer observation, reassessment, and preventive nursing attention, it inherently creates ambiguity between actual clinical diagnosis and elevated risk. Consequently, this methodological choice may impact the model’s diagnostic validity and introduce label noise, as the algorithm predicts the need for surveillance rather than the true physiological onset of a complication. Therefore, when interpreting the predictive performance, it is crucial to recognize that the model’s primary utility lies in early risk stratification and guiding proactive nursing interventions, rather than serving as a definitive diagnostic tool.

From a nursing perspective, the main contribution of this study is to connect risk prediction with nurse-actionable surveillance. In postoperative care, risk scores have limited practical value unless they help nurses prioritize what to observe, when to escalate, and how to respond. The structured action–evidence–rationale format was designed to reduce the gap between algorithmic output and bedside judgment. Thus, the practical significance of this system should ultimately be judged by whether it improves nurses’ situational awareness, prioritization, and consistency of care, rather than by technical performance alone.

At the same time, the recommendation space in the present study was intentionally restricted to 12 predefined nursing surveillance actions to maintain interpretability and reduce unsafe variability in LLM generation. However, these actions should be understood as a standardized core surveillance framework rather than an exhaustive representation of postoperative nursing care. In actual practice, nursing responses are often more nuanced and may vary according to surgical subtype, respiratory severity, comorbidity burden, institutional protocol, and evolving patient status. Future work should therefore examine whether finer-grained subcategories, context-sensitive triggering logic, or hierarchical refinement can better capture clinical complexity without sacrificing usability.

The LLM-based recommendation module also requires careful interpretation. Although supervised fine-tuning improved the correctness of recommended actions, LLM outputs remain probabilistic and may still generate generic, inconsistent, or weakly justified suggestions. In real clinical settings, additional concerns include protocol misalignment, auditability, version control, privacy protection, and accountability for erroneous recommendations [24]. For these reasons, LLM-generated outputs should be regarded as assistive recommendations under clinical oversight, not autonomous directives.

The current evaluation also remains limited. Although expert review supported the feasibility of the recommendation module, the number of evaluated cases was small and the assessment relied primarily on expert judgment. Such evaluation is useful for initial feasibility testing, but it is insufficient to establish robust evidence of recommendation reliability or clinical effectiveness. Future studies should therefore incorporate larger and more diverse case sets, structured scoring rubrics, inter-rater agreement analysis, error-type analysis, and prospective assessment under real clinical conditions. In addition, implementation-oriented metrics, such as alert acceptance, action uptake, time to escalation, and user trust, may provide a more meaningful assessment of utility than correctness alone.

A further practical issue is how this system can be integrated into real clinical workflows. The usefulness of recommendation outputs depends not only on their content, but also on when, where, and how they are presented during care delivery. If poorly integrated, the system may increase documentation burden, duplicate decision steps, or contribute to alert fatigue rather than reduce workload. Conversely, if appropriately embedded into existing EMR interfaces and escalation pathways, it may help nurses focus attention on high-risk patients, support prioritization, and improve consistency of surveillance. Accordingly, future prospective studies should evaluate workflow fit, response burden, usability, and the balance between cognitive support and additional task load in actual ward settings.

Beyond inpatient use, prior CDSS and remote monitoring studies suggest that postoperative risk management may increasingly extend into post-discharge surveillance and resource allocation [25,26,27]. However, whether the present framework can be expanded in this way remains uncertain and would depend on stable data capture, workflow integration, and validation across settings [25,26,27].

Several limitations should be acknowledged. First, this was a single-center retrospective study without external validation. Second, nursing surveillance variables may partly reflect documentation practice rather than patient physiology alone, and EMR-based models may absorb site-specific biases that affect fairness and generalizability [28]. Third, the recommendation module was not prospectively evaluated in real workflows, and the current expert review remains limited in scale and methodological rigor. Fourth, the predefined 12-action set, while intentionally designed to preserve consistency and interpretability, may not fully capture the breadth and granularity of actual nursing interventions. Finally, the current study did not assess calibration drift, subgroup consistency, alert burden, or implementation outcomes, all of which are important for responsible deployment [22,23,24].

Future research should therefore focus on multicenter external validation, evaluation across heterogeneous EMR environments, prospective implementation studies with usability and safety endpoints, refinement of evaluation frameworks for recommendation quality, and further development of recommendation generation to improve consistency, traceability, workflow compatibility, and alignment with institutional nursing protocols [22,23,24]. Through these steps, PPC prediction may evolve from a technically promising model into a more transferable, trustworthy, and clinically meaningful nursing-centered decision support system.

## 5. Conclusions

This study demonstrated the feasibility of a holistic, nursing-centered clinical decision support system (CDSS) for clinically significant postoperative pulmonary complications (PPCs) in abdominal surgery patients by integrating prediction, explanation, and intervention into a unified pipeline. Using single-center retrospective EMR data, the proposed model achieved solid discriminative performance (AUROC 0.810) while maintaining a balanced precision–recall profile through imbalance-aware training and decision-threshold optimization. Beyond risk prediction, the system translated model outputs into structured nursing surveillance recommendations with supporting evidence and rationale, and supervised fine-tuning improved recommendation correctness in expert evaluation. These findings highlight the potential value of linking PPC risk estimation with nurse-actionable surveillance support rather than limiting the system to prediction alone.

At the same time, the findings should be interpreted within the scope of the present study. Because the model was developed using retrospective data from a single institution, further validation is needed to confirm generalizability across different hospitals, EMR environments, and clinical workflows. In addition, although the LLM-based recommendation module showed improved performance after supervised fine-tuning, continued refinement will be important to ensure consistency, evidence traceability, and alignment with institutional protocols in real-world settings.

Overall, this study suggests that integrating PPC risk prediction with nursing surveillance signals and structured recommendations may enhance the clinical relevance and usability of postoperative decision support. Future research should focus on multicenter external validation, prospective workflow-based evaluation, calibration and thresholding strategies tailored to surveillance capacity, and further development of recommendation generation for robust and trustworthy implementation.

## Figures and Tables

**Table 1:** Data Feature Description.

Feature	Data Type	Description
Age	Numeric	Age (years)
Age_1	Categorical	Age group category
Gender	Categorical	Sex (M/F)
Surgical_code_site	Categorical	Surgical site/organ category
Sugical_code_approach	Categorical	Surgical approach
Diagnostic_code	Categorical	Primary diagnosis category
Anesthesia_time	Numeric	Anesthesia duration (minutes)
Anesthesia_time_1	Categorical	Anesthesia time category
Is_emergency	Categorical	Admission route
Stature	Numeric	Height (assumed cm)
Weight	Numeric	Body weight (assumed kg)
BMI	Numeric	Body mass index (assumed kg/m^2^)
BMI_1	Categorical	BMI binned/grade code
Is_smoke	Categorical	Smoking status
Is_drinker	Categorical	Alcohol use
Is_acivity_free	Categorical	Activity/functional status
L_tube	Categorical	L-tube insertion
Hb_min	Numeric	Minimum hemoglobin
SBP_max	Numeric	Maximum systolic blood pressure
SBP_min	Numeric	Minimum systolic blood pressure
DBP_max	Numeric	Maximum diastolic blood pressure
DBP_min	Numeric	Minimum diastolic blood pressure
BT_max	Numeric	Maximum body temperature
BT_min	Numeric	Minimum body temperature
RR_max	Numeric	Maximum respiratory rate
RR_min	Numeric	Minimum respiratory rate
PR_max	Numeric	Maximum pulse rate
PR_min	Numeric	Minimum pulse rate
Min_SpO_2_	Numeric	Lowest SpO_2_
SBP_judge	Bool	Abnormal SBP flag
DBP_judge	Bool	Abnormal DBP flag
BT_judge	Bool	Abnormal temperature flag
RR_judge	Bool	Abnormal respiratory rate flag
PR_judge	Bool	Abnormal pulse flag
SpO_2__judge	Bool	Abnormal SpO_2_ flag
NRS_judge	Bool	Abnormal pain flag
Has_medical_history	Categorical	Comorbidity/medical history code
NRS_avg	Numeric	Average pain score
Systemic_infection_symptoms	Bool	Systemic infection symptoms present
Bronchial_secretion_symptoms	Bool	Bronchial secretion/sputum-related symptoms present
Respiratory_symptoms	Bool	Respiratory symptoms present

BMI = body mass index, Hb = hemoglobin, L-tube = Levin tube, SBP = systolic blood pressure, DBP = diastolic blood pressure, BT = body temperature, RR = respiratory rate, PR = pulse rate, SpO_2_ = saturation of percutaneous oxygen, and NRS = numerical rating scale.

**Table 2:** Nursing Surveillance Actions.

Action
1. Monitor the respiratory rate and breathing pattern
2. Monitor cough and breathing discomfort
3. Monitor the characteristics of the sputum
4. Monitor oxygen saturation (SpO_2_)
5. Verify intervals for vital signs and SpO_2_ monitoring
6. Monitor body temperature
7. Monitor symptoms: heat sensation, chills, shivering, facial flushing
8. Monitor the level and pattern of pain
9. Encourage the patient to express their pain
10. Explain the importance of early ambulation and provide encouragement
11. Communicate with the doctor about changes in patient status
12. Communicate with the doctor about the necessity of a follow-up chest X-ray

SpO_2_ = saturation of percutaneous oxygen.

**Table 3:** Experimental Results.

Methods	Models	Precision	Recall	Accuracy	F1-Macro	ROC-AUC	PR-AUC	Specificity
**ML-based**	RF	0.466 [95% CI 0.441–0.494]	0.553 [95% CI 0.523–0.579]	0.775 [95% CI 0.763–0.785]	0.680 [95% CI 0.665–0.694]	0.785 [95% CI 0.771–0.800]	0.495 [95% CI 0.464–0.522]	0.833 [95% CI 0.822–0.844]
	LR	0.473 [95% CI 0.432–0.482]	0.578 [95% CI 0.541–0.595]	0.774 [95% CI 0.758–0.779]	0.682 [95% CI 0.664–0.692]	0.781 [95% CI 0.767–0.795]	0.493 [95% CI 0.460–0.519]	0.825 [95% CI 0.810–0.833]
	XGB	0.465 [95% CI 0.428–0.478]	0.529 [95% CI 0.490–0.545]	0.774 [95% CI 0.758–0.779]	0.673 [95% CI 0.653–0.681]	0.777 [95% CI 0.763–0.791]	0.485 [95% CI 0.453–0.512]	0.838 [95% CI 0.825–0.845]
	LGBM	0.451 [95% CI 0.418–0.470]	0.532 [95% CI 0.473–0.528]	0.764 [95% CI 0.754–0.775]	0.663 [95% CI 0.646–0.674]	0.763 [95% CI 0.748–0.777]	0.472 [95% CI 0.440–0.501]	0.824 [95% CI 0.823–0.844]
**DL-based**	Tabular MLP	0.471 [95% CI 0.444–0.499]	0.513 [95% CI 0.483–0.539]	0.778 [95% CI 0.768–0.789]	0.675 [95% CI 0.659–0.688]	0.774 [95% CI 0.759–0.790]	0.496 [95% CI 0.467–0.525]	0.848 [95% CI 0.839–0.859]
	FT Transformer	0.445 [95% CI 0.423–0.470]	0.615 [95% CI 0.586–0.643]	0.759 [95% CI 0.749–0.770]	0.678 [95% CI 0.665–0.692]	0.783 [95% CI 0.769–0.797]	0.497 [95% CI 0.469–0.527]	0.798 [95% CI 0.786–0.810]
	TabNet	0.441 [95% CI 0.418–0.468]	0.494 [95% CI 0.467–0.520]	0.764 [95% CI 0.754–0.774]	0.657 [95% CI 0.644–0.671]	0.741 [95% CI 0.727–0.756]	0.430 [95% CI 0.404–0.458]	0.835 [95% CI 0.825–0.845]
**Ours**		**0.544** **[95% CI 0.484–0.608]**	**0.546** **[95% CI 0.489–0.606]**	**0.810** **[95% CI 0.789–0.832]**	**0.712** **[95% CI 0.681–0.742]**	**0.810** **[95% CI 0.782–0.839]**	**0.531** **[95% CI 0.470–0.596]**	**0.879** **[95% CI 0.859–0.900]**

DL = deep learning, FT = feature tokenizer, LGBM = light gradient boosting machine, LR = logistic regression, ML = machine learning, MLP = multilayer perceptron, RF = random forest, ROC-AUC = receiver operating characteristic-area under curve, PR-AUC = Precision-Recall Area Under the Curve, XGB = extreme gradient boost, and CI = Confidence interval.

**Table 4:** Ablation Study.

Methods	Precision	Recall	Accuracy	F1-Macro	ROC-AUC	PR-AUC	Specificity
Ours	**0.54** **4**	0.546	**0.81** **0**	**0.71** **2**	**0.810**	**0.530**	**0.879**
w/o SHAP Importance	0.508	0.515	0.795	0.691	0.791	0.470	0.868
w/o Imbalance	0.506	0.508	0.794	0.688	0.794	0.509	0.869
Baseline	0.494	0.496	0.789	0.681	0.800	0.498	0.866

SHAP = Shapley additive explanations, w/o = without.

**Table 5:** Human Evaluation.

Model	Score (5)	Score (4)	Score (3)	Score (2)	Score (1)	Total
Base	52	21	19	8	0	100
Ours	**79**	**12**	**9**	**0**	**0**	**100**

**Table 6:** Case study.

**Patient** **information**	Hb_min: 11.7 SBP_max: 151 SBP_min: 113 DBP_max: 130 DBP_min: 58 BT_max: 37.1 BT_min: 36.4 RR_max: 20 RR_min: 20 PR_max: 64 PR_min: 46 Min_SpO_2_: 95 NRS_avg: 2.125 systemic_infection_symptoms: FALSE bronchial_secretion_symptoms: FALSE respiratory_symptoms: FALSE		
**Action** **list**	1. Monitor the respiratory rate and breathing pattern		
	2. Monitor cough and breathing discomfort		
	3. Monitor the characteristics of the sputum		
	4. Monitor oxygen saturation (SpO_2_)		
	5. Verify intervals for vital signs and SpO_2_ monitoring		
	6. Monitor body temperature		
	7. Monitor symptoms: heat sensation, chills, shivering, facial flushing		
	8. Monitor the level and pattern of pain		
	9. Encourage the patient to express their pain		
	10. Explain the importance of early ambulation and provide encouragement		
	11. Communicate with the physician about changes in patient status		
	12. Communicate with the doctor about the necessity of a follow-up chest X-ray		
**Methods**	Ours	Base	Base
**Actions**	Verify intervals for vital signs and SpO_2_ monitoring	Monitor body temperature	Monitor the respiratory rate and breathing pattern
**Evidence**	Blood Pressure (SBP): 151 mmHg	Body Temperature: 37.1 °C	Blood pressure (SBP): 151 mmHg
**Rationale**	Elevated blood pressure requires monitoring and potential physician notification.	Baseline temperature should be documented.	Elevated blood pressure can be associated with respiratory distress.
**Label**	**✓** (Correct)	**✕** (Unnecessary action)	**✕** (Evidence mismatch)

BT = body temperature, DBP = diastolic blood pressure, Hb = hemoglobin, NRS = numerical rating scale, PR = pulse rate, RR = respiratory rate, SBP = systolic blood pressure, and SpO_2_ = saturation of percutaneous oxygen.

## Data Availability

Restrictions apply to data availability. Data were obtained from the Samsung Changwon Hospital and are available from its clinical data warehouse, with institutional permission. Due to the use of hospital clinical data, the dataset is not publicly available.

## Supplementary Materials

The following supporting information can be downloaded at https://www.mdpi.com/article/10.3390/healthcare14081083/s1: Table S1: Patient, Surgical, Vital-Sign, and Nursing-Note–Derived Features Compared Between PPCs and Non-PPCs Groups.

## Appendix A

In the Appendix A, it contains content related to the prompt used for the LLM to present recommended actions in the nursing surveillance decision-support system. Table A1 is the full text of the prompt used. Regarding the role of the LLM, it is a system that supports nursing surveillance, and it was assigned the role of presenting recommended actions when patient information is abnormal or PPCs are identified. In the Operation rules, it presents the rule that the LLM should select and present actions only from among the 12 actions for patients whose underlying information is abnormal, and that it should not present any actions otherwise. The Action List consists of 12 standard actions that the LLM recommends based on the information it receives, and the Mapping Logic is the reference criterion when matching actions with patient information. Finally, the Output Format defines the format of the nursing surveillance support output presented by the LLM.

**Table A1:** Nursing Surveillance Decision-making Support Generation Prompt.

**Role**	1. You are a highly precise Nursing Surveillance Support System. 2. Your ONLY goal is to generate nursing actions for ABNORMAL conditions and RISK FACTORS.
**Operational** **Rules**	1. FOCUS ON SECTION A ONLY: You must generate actions ONLY based on the items listed in “SECTION A: ISSUES REQUIRING ATTENTION”. 2. IGNORE SECTION B: Items in ”SECTION B: NORMAL FINDINGS” are provided for context only. NEVER generate actions for them. 3. MANDATORY ACTION: Since “PPC Risk Label: TRUE” is always in Section A, you MUST always recommend “Educate on importance of early ambulation…”.
**Action** **List**	1. “Monitor the respiratory rate and breathing pattern” 2. “Monitor cough and breathing discomfort” 3. “Monitor the characteristics of the sputum” 4. “Monitor oxygen saturation (SpO_2_)” 5. “Verify intervals for vital signs and SpO_2_ monitoring” 6. “Monitor body temperature” 7. “Monitor symptoms: heat sensation, chills, shivering, facial flushing” 8. “Monitor the level and pattern of pain” 9. “Encourage the patient to express their pain” 10. “Explain the importance of early ambulation and provide encouragement” 11. “Communicate with the physician about changes in patient status” 12. “Communicate with the doctor about the necessity of a follow-up chest X-ray”
**Mapping** **Logic**	1. High Risk (Label = True) → Action 10 (MANDATORY) 2. Low SpO_2_ (<95%) → Actions 4, 5. (Severe? Add 11) 3. Abnormal RR (<12 or >20) → Actions 1, 2. 4. Fever (>37.5) → Actions 6, 7. 5. Pain (NRS ≥ 4) → Actions 8, 9. 6. Sputum/Cough → Actions 2, 3. 7. Multiple Abnormalities ** → Action 11. 8. Critical Distress → Action 12 (X-ray).
**Output** **Format**	Return ONLY a valid JSON list. [ { “Action”: “Exact string from the list”, “Evidence”: “The specific abnormal value or risk factor from SECTION A”, “Rationale”: “Why this action is necessary.” } ]
