# Supplementary material for: A Holistic Nursing Surveillance Decision Support System for Postoperative Pulmonary Complications After Abdominal Surgery: A Retrospective Cohort Study
Source: Healthcare (Basel). 2026 Apr 18;14(8):1083. doi: 10.3390/healthcare14081083 (PMC13116495; doi:10.3390/healthcare14081083)
Supplement: Supplementary file 1 [file healthcare-14-01083-s001.zip › healthcare-4214233-supplementary.pdf]

Table S1. Patient, Surgical, Vital-Sign, and Nursing-Note–Derived Features Compared Between PPCs and Non-PPCs Groups.

Feature	Total (N=6,285)	No PPC (N=4,974)	PPC (N=1,311)	p-value
Age (years)	56.7 ± 14.6	55.0 ± 14.4	63.1 ± 13.5	<0.001
Anesthesia duration (min)	118.1 ± 80.4	108.0 ± 72.2	156.4 ± 96.6	<0.001
Height (cm)	164.3 ± 8.7	164.4 ± 8.7	164.1 ± 8.9	0.353
Weight (kg)	65.8 ± 12.7	65.5 ± 12.5	66.9 ± 13.4	<0.001
BMI (kg/m^2)	24.3 ± 3.6	24.1 ± 3.6	24.7 ± 3.8	<0.001
Minimum hemoglobin (g/dL)	11.5 ± 1.8	11.7 ± 1.7	10.8 ± 2.1	<0.001
Maximum SBP (mmHg)	141.6 ± 16.4	139.8 ± 16.2	148.1 ± 15.6	<0.001
Minimum SBP (mmHg)	106.5 ± 10.9	106.7 ± 10.7	105.6 ± 11.8	0.001
Maximum DBP (mmHg)	86.2 ± 9.6	85.3 ± 9.5	89.4 ± 9.5	<0.001
Minimum DBP (mmHg)	61.1 ± 8.2	61.4 ± 8.0	59.8 ± 8.5	<0.001
Maximum Body Temperature (°C)	37.4 ± 0.5	37.3 ± 0.4	37.6 ± 0.6	<0.001
Minimum Body Temperature (°C)	36.4 ± 0.3	36.4 ± 0.3	36.3 ± 0.4	<0.001
Maximum Respiratory Rate (/min)	20.6 ± 3.4	20.4 ± 3.3	21.1 ± 3.6	<0.001
Minimum Respiratory Rate (/min)	19.0 ± 1.3	19.1 ± 1.2	18.9 ± 1.5	<0.001
Maximum Pulse Rate (/min)	89.2 ± 13.3	87.3 ± 12.0	96.2 ± 15.3	<0.001
Minimum Pulse Rate (/min)	63.5 ± 7.6	63.3 ± 7.5	64.3 ± 7.9	<0.001
Lowest SpO_2 (%)	94.6 ± 2.2	95.0 ± 1.8	93.3 ± 3.1	<0.001
Average pain score (NRS)	2.4 ± 0.5	2.4 ± 0.5	2.4 ± 0.5	0.560
Age group category				<0.001
- older_adult	2311 (36.8%)	1848 (37.2%)	463 (35.3%)	
- elderly	1975 (31.4%)	1347 (27.1%)	628 (47.9%)	
- middle_aged	1786 (28.4%)	1580 (31.8%)	206 (15.7%)	
- young_adult	213 (3.4%)	199 (4.0%)	14 (1.1%)	
Sex				<0.001
- M	3466 (55.1%)	2620 (52.7%)	846 (64.5%)	
- F	2819 (44.9%)	2354 (47.3%)	465 (35.5%)	
Surgical site/organ category				<0.001
- gallbladder	4929 (78.4%)	4108 (82.6%)	821 (62.6%)	
- stomach	1049 (16.7%)	710 (14.3%)	339 (25.9%)	
- liver	243 (3.9%)	115 (2.3%)	128 (9.8%)	
- other	64 (1.0%)	41 (0.8%)	23 (1.8%)	
Surgical approach				<0.001
- laparoscopic	4787 (76.2%)	3994 (80.3%)	793 (60.5%)	
- open_surgery	1498 (23.8%)	980 (19.7%)	518 (39.5%)	
Primary diagnosis category				<0.001
- gallbladder_disease	4780 (76.1%)	3986 (80.1%)	794 (60.6%)	
- gastric_cancer	925 (14.7%)	628 (12.6%)	297 (22.7%)	
- other_disease	347 (5.5%)	245 (4.9%)	102 (7.8%)	
- hepatobiliary_pancreatic_cancer	233 (3.7%)	115 (2.3%)	118 (9.0%)	
Anesthesia time category				<0.001

- <180_min	5030 (80.0%)	4177 (84.0%)	853 (65.1%)	
- >=180_min	1255 (20.0%)	797 (16.0%)	458 (34.9%)	
Admission route				<0.001
- outpatient	4716 (75.0%)	3910 (78.6%)	806 (61.5%)	
- emergency_room	1569 (25.0%)	1064 (21.4%)	505 (38.5%)	
BMI category				<0.001
- <18.5	225 (3.6%)	180 (3.6%)	45 (3.4%)	
- 18.5 ~ 22.9	2096 (33.3%)	1725 (34.7%)	371 (28.3%)	
- 23 ~ 24.9	1588 (25.3%)	1282 (25.8%)	306 (23.3%)	
- >=25	2376 (37.8%)	1787 (35.9%)	589 (44.9%)	
Smoking status				<0.001
- no	4588 (73.0%)	3683 (74.0%)	905 (69.0%)	
- yes_current_smoker	1158 (18.4%)	902 (18.1%)	256 (19.5%)	
- yes_former_smoker	539 (8.6%)	389 (7.8%)	150 (11.4%)	
Alcohol use				0.074
- no	4255 (67.7%)	3340 (67.1%)	915 (69.8%)	
- yes	2030 (32.3%)	1634 (32.9%)	396 (30.2%)	
Activity/functional status				<0.001
- independent	6223 (99.0%)	4941 (99.3%)	1282 (97.8%)	
- restricted	62 (1.0%)	33 (0.7%)	29 (2.2%)	
L-tube insertion				<0.001
- no	5763 (91.7%)	4645 (93.4%)	1118 (85.3%)	
- yes	522 (8.3%)	329 (6.6%)	193 (14.7%)	
Comorbidity/medical history code				<0.001
- None	1374 (21.9%)	1188 (23.9%)	186 (14.2%)	
- Hypertension	340 (5.4%)	257 (5.2%)	83 (6.3%)	
- Diabetes	101 (1.6%)	70 (1.4%)	31 (2.4%)	
- Other	2484 (39.5%)	2042 (41.1%)	442 (33.7%)	
- Hypertension & Diabetes	125 (2.0%)	92 (1.8%)	33 (2.5%)	
- Hypertension & Other	1026 (16.3%)	750 (15.1%)	276 (21.1%)	
- Diabetes & Other	302 (4.8%)	210 (4.2%)	92 (7.0%)	
- Hypertension & Diabetes & Other	533 (8.5%)	365 (7.3%)	168 (12.8%)	
Abnormal SBP flag (yes)	3707 (59.0%)	2695 (54.2%)	1012 (77.2%)	<0.001
Abnormal DBP flag (yes)	2572 (40.9%)	1845 (37.1%)	727 (55.5%)	<0.001
Abnormal temperature flag (yes)	775 (12.3%)	435 (8.7%)	340 (25.9%)	<0.001
Abnormal respiratory rate flag (yes)	963 (15.3%)	589 (11.8%)	374 (28.5%)	<0.001
Abnormal pulse flag (yes)	937 (14.9%)	527 (10.6%)	410 (31.3%)	<0.001
Abnormal SpO_2 flag (yes)	896 (14.3%)	473 (9.5%)	423 (32.3%)	<0.001
Abnormal pain flag (yes)	2004 (31.9%)	1435 (28.9%)	569 (43.4%)	<0.001
Systemic infection symptoms present (yes)	758 (12.1%)	399 (8.0%)	359 (27.4%)	<0.001
Bronchial secretion symptoms present (yes)	61 (1.0%)	18 (0.4%)	43 (3.3%)	<0.001
Respiratory symptoms present (yes)	121 (1.9%)	60 (1.2%)	61 (4.7%)	<0.001

BMI = body mass index, Hb = hemoglobin, L-tube = Levin tube, SBP = systolic blood pressure, DBP = diastolic blood pressure, BT = body temperature, RR = respiratory rate, PR = pulse rate, SpO2 = saturation of percutaneous oxygen, NRS = numerical rating scale.
